# A Review of Floating Photovoltaic Systems: Prospects, Challenges, and Sustainability Considerations

**DOI:** 10.1002/gch2.202500581

**Published:** 2026-02-17

**Authors:** Moslema Hoque Oeishee, Md. Mosaddequr Rahman

**Affiliations:** ^1^ Department of Electrical and Electronic Engineering BSRM School of Engineering BRAC University Dhaka Bangladesh

**Keywords:** anchoring and mooring, environment, floating PV, FPV, life cycle analysis, policy, sustainability

## Abstract

The floating photovoltaic (FPV) system is gaining global attention as a promising renewable energy solution that addresses both land scarcity and rising energy demand while contributing to climate change mitigation. Unlike conventional ground‐mounted solar, FPV installations make use of reservoirs, lakes, and coastal waters, which not only saves valuable land but also improves efficiency through natural cooling, resulting in 10%–20% higher energy yields and up to 70% reduction in water evaporation. This paper reviews FPV's evolution from early concepts in the late 20th century to rapid commercialization after 2007. The study outlines three main system designs: Pontoon‐based, flexible membrane, and submerged structures, alongside critical components such as floating platforms, mooring systems, and AI‐enabled monitoring that ensure stability and performance. Engineering strategies discussed in this work include optimized site selection, hydrodynamic resilience, and auxiliary measures like cooling and automated cleaning. The paper also evaluates environmental interactions and shows that FPV may alter aquatic ecosystems by reducing light penetration and dissolved oxygen levels. However, it also demonstrates that these risks can be mitigated through eco‐friendly anchoring and limited coverage. Life cycle assessments presented here highlight sustainability benefits, including energy payback times of 1–3 years, emission reductions of 5%–10%, and recyclability rates up to 90%. Asia leads in current deployment, where the planned capacity of the largest FPV project has surpassed 2200 MW. Despite the availability of several FPV technologies, their extensive development is hindered by inadequate policy support as well as challenges such as material durability, maintenance complexity, and fragmented regulations. However, FPV offers an opportunity to combine clean energy generation with sustainable water management. This paper provides insights for policymakers to design supportive regulations, for engineers to enhance system reliability, and for researchers to explore innovations, thereby guiding all stakeholders in advancing FPV as a key driver of sustainable and resilient energy transitions.

## Graphical abstract

This review synthesizes recent advances in floating photovoltaic (FPV) systems by integrating technology classification, engineering design, aquatic ecosystem interactions, and sustainability assessment. The image illustrates the conceptual framework linking FPV configurations, mooring strategies, thermal and ecological effects, and life cycle outcomes, providing a unified perspective to support environmentally responsible FPV deployment and future research.

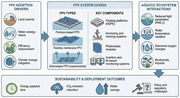

**Abbreviations**
AC: alternating current
AI: artificial intelligence
API: American Petroleum Institute
CdCl_2_
: cadmium chloride
CO_2_
: carbon dioxide
DC: direct current
DO: dissolved oxygen
EIA: environmental impact assessment
EPBT: energy payback time
EROI: energy return on investment
FEM: finite element modeling
FPV: floating photovoltaic
GFRP: glass‐fiber‐reinforced plastic
GIS: geographic information systems
GW: Gigawatt
HDPE: high‐density polyethylene
IPCC: intergovernmental panel on climate change
kW: Kilowatt
LCA: life cycle assessment
MDPE: medium‐density polyethylene
MgCl_2_
: magnesium chloride
ML: machine learning
MW: Megawatt
PAR: photosynthetically active radiation
PV: photovoltaic
PVsyst: photovoltaic system simulation software
RETScreen: renewable energy technology screen
SAM: system advisor model
SCADA: supervisory control and data acquisition
TW: Terawatt
USD: United States Dollar

## 1 Introduction

### 1.1 Background on Renewable Energy Transition

Global warming is one of the leading causes of climate change, which is among the most significant issues of the modern world. Despite their long‐time role in the development of the industry, fossil fuels such as coal, oil, and natural gas are now the primary causes of CO_2_ emissions and global warming, and deteriorating air quality [1]. According to the IPCC, a rise in temperature beyond 1.5°C above the levels before the industrial age will lead to irreversible consequences, including the collapse of the ecosystem, severe weather, and flooding [2]. Despite global efforts like the Paris Agreement, the trajectory toward staying below 2 °C remains insufficient [3]. Fossil fuels are very harmful to the environment and human health. Research reports that health expenses relating to climate change are likely to run high at USD 4 billion annually by 2030 [4, 5, 6]. The lack of access to electricity, especially in the low‐ and middle‐income regions, further contributes to social and economic inequality because over 1.2 billion individuals today do not have regular access to it [7]. This kind of unequal distribution of energy impairs the sustainability development objectives and worsens socioeconomic disparities.

Emissions and energy disparity are two issues that are making the world move faster toward renewable energy technologies (RETs), including solar, wind, hydro, and biomass. The main characteristic of solar photovoltaics (PV) is that it is completely emission free, cost effective and can be scaled in modules [8, 9]. This has resulted in a rapid increase in solar PV capacity of the world, with 40 GW of solar PV capacity in the year 2010 to over 1, 200 GW in the year 2023 with a Compound Annual Growth Rate (CAGR) of more than 30 [10]. These increases have been caused by technological advancements, efficient laws, lowering of the price of PV modules, and more awareness of the environment [11, 12]. PV assists in driving energy security and climate targets both in industrialised and developing countries. Whereas the developing world is utilizing PV to augment the power supply in distant areas [14], the developed world is investing in solar to meet decarbonization targets [13]. As a result, PV is essential for promoting environmental justice and public health in addition to technological growth [15, 16]. In the broader framework, FPV systems are emerging as a cutting‐edge area of solar technology that could help resolve some land and efficiency concerns, which will be covered in the review's subsequent paragraphs.

### 1.2 Role of Solar PV and Emergence of Floating PV

Solar PV technology is revolutionizing global energy systems, with installed capacity surpassing 2.2 terawatts (TW) and annual additions exceeding 600 GW as of 2024 [17, 18]. Rising energy consumption, favorable government policies, and falling PV module costs are the primary causes of this impressive increase [19]. PV systems have developed into a variety of shapes to satisfy a range of land, environmental, and infrastructure constraints. These forms mainly include ground‐mounted and rooftop systems, with innovations such as canal‐top and offshore installations [20, 21]. The most frequently used ground‐mounted photovoltaic system that is used in utility‐scale applications is pole, foundation, and ballasted mount designs; the latter is particularly well suited in sensitive or limited locations [22, 23, 24]. Having power outputs in the 5 to over 100 kW range, rooftop PV systems are used mainly in urban locations as a process of decentralised power production [25, 26]. Nonetheless, the scale and orientation of the roof inhibit their performance [27, 28]. According to the example of the Narmada canal project in India, where it consumes 9 million litres of water annually and generates 1 MW of power, novel solutions, like the canal top PV, not only can generate electricity, but also can save water by reducing evaporation by 30%–40% [29, 30, 31, 32]. By leveraging existing infrastructure, canal‐top PV reduces grid and construction costs, offering a scalable solution for countries like India, which targets 100 MW under its National Solar Mission [33]. Offshore PV systems leverage the ocean’s vast solar potential estimated at approx.173,000 TW and provide a viable solution for coastal regions, where 40% of the global population resides [34, 35, 36]. Offshore photovoltaic system is an effective solution for coastal locations, which house 40% of the world population; this is Natural cooling in offshore platforms leads to a 5 to15% increase in PV efficiency, and other novel materials, such as magnesium chloride (MgCl_2_), can decrease the cost of panels by up to 25% [37, 38]. PV offshore platforms have demonstrated the scalability of offshore PV and its potential to power the marine industry [39].

**FIGURE 1:**
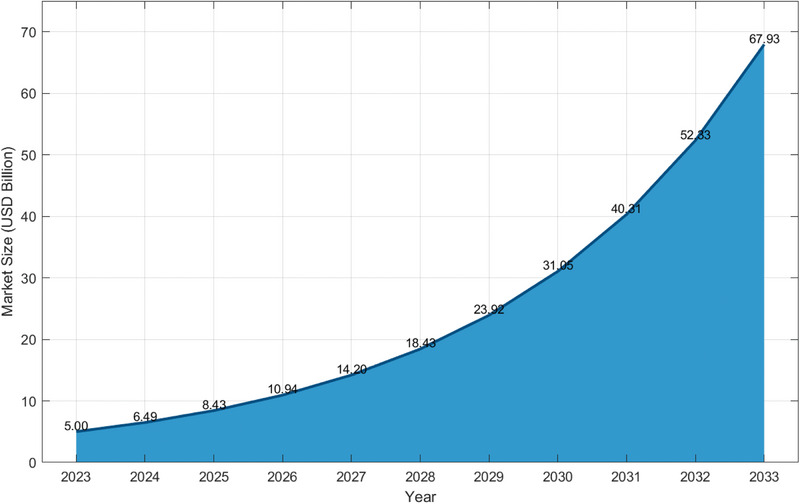
Global floating solar PV market size forecast (2024–2033) data source: Nova one advisor [10]. Copyright the Authors, 2025.

Despite these advancements, conventional PV systems still face challenges related to land use, environmental impact, and heat‐induced efficiency losses [40]. Floating PV systems offer an alternative to land and efficiency challenges by using water surfaces like lakes and reservoirs [41]. Countries with dense populations, such as Japan, Korea, and Singapore, are increasingly turning to FPV systems as a viable alternative to land‐based solar, driven by both spatial constraints and environmental considerations [42, 43, 44]. They stay cooler, boosting efficiency by 10%–15%, and help reduce water evaporation and algae growth [45, 46, 47]. Consequently, there has been tight growth in the past years in the FPV market because of these advantages. Its market size across the world totaled USD 5.0 billion in 2023 and is presently increasing to USD 67.93 billion by 2033 at an astonishing compound annual growth rate (CAGR) of 29.81% that is shown in Figure 1 [10]. The greatest number of installed projects are found in Asia‐Pacific, especially in China, Japan, India, and South Korea, where scarcity of land and government incentives have boosted use [48].

FPV also holds offshore potential: oceans receive 70% of global solar irradiance [49], and more than half the global population lives within 100 km of coastlines [50]. These systems can supply coastal and marine infrastructure, including ports, desalination facilities, and fish farms, while minimizing transmission losses [51]. These potential outcomes highlight how FPV systems might promote environmental sustainability and diversify energy supply sources. FPV provides a special set of advantages in terms of cost, technology, and the environment as compared to ground‐mounted PV systems. Table 1 demonstrates that floating PV is less impacted by soiling, enjoys the benefit of water reflection to improve bifacial module efficiency, and suffers little from high temperatures, however bird droppings may be a local problem. Ground‐mounted PV faces issues like heat loss, soiling, shading, land use, and habitat disruption. While FPV saves land and water, it involves higher structural costs and regulatory challenges [52].

**TABLE 1:** Comparison between floating photovoltaic and ground‐mounted photovoltaic systems [53].

Feature	Floating PV system	Ground‐mounted PV systems
Technology readiness	More than 350 operational installations globally	Over 1000 systems have been deployed worldwide
Energy performance	Minimal temperature‐related losses; efficient bifacial performance; water cooling effect; low soiling	High temperature losses; albedo‐dependent; significant soiling; fixed, rigid structure
Regulatory framework	Regulatory uncertainty; unclear ownership over water bodies	Well‐defined and standardized regulatory pathways
Cost factors	Reduced site development costs; higher anchoring/floating platform costs	Higher capital from land acquisition; relatively lower structure cost
Environmental benefits	Reduces evaporation and improves water conservation	Potential loss of productive land and biodiversity
Installation process	Easier deployment on calm water surfaces	Depends heavily on terrain quality and site grading
Standards and testing	Lack of unified international testing standards	Globally accepted standards in place

FPV adapts to a variety of environments and gets over traditional solar constraints. It is an essential component of upcoming renewable energy systems because of its efficiency‐boosting hybridization with hydropower [54]. As a logical extension of conventional solar photovoltaics in response to the overlapping forces of land accessibility, water resource control, and climate stability, FPV systems have emerged as a strategic area of concern in recent years. The ground‐mounted PV is fast spreading worldwide; it has increased competition with land use in agriculture, urbanization, and in the conservation of ecosystem especially in highly populated and water‐abundant parts of Asia and Europe. Here, FPV is particularly an interesting substitute, as it opens the opportunity to place solar facilities on waters with scanty utilization like reservoirs, irrigation ponds, and hydropower dams, thus uncoupling solar growth to terrestrial land‐use limitations [42, 47, 52].

The growth in FPV development is now being presented as part of the larger water‐energy climate nexus than spatial efficiency. FPV systems can provide co‐benefits of energy and water delivery through waters used to deploy FPVs, which are usually part of a drinking water supply system, irrigation, flood management, or hydropower. The empirical research has revealed that partial surface coverage with FPV has the capacity to lower evaporation of reservoirs, regulate surface water temperatures, and inhibit exponential expansion of algae, and the closeness to water improves the photovoltaic performance due to cooling influences [45,^46^]. These compound benefits are especially applicable in climatic conditions, which will be characterised by an increase in temperature, evaporation losses, and pressure on freshwater resources.

Recent FPV growth has also been determined by policy and market forces as well. National energy security, net‐zero, and renewable energy goals have encouraged governments to concentrate on novel solar deployment strategies that minimize social and environmental trade‐offs. Several countries in Asia and Europe have explicitly incorporated floating solar into renewable energy roadmaps and demonstration programs, reflecting growing institutional confidence in the technology's scalability and sustainability potential [42].

Technologically, one should be able to differentiate photovoltaic material processing concerns and FPV system‐level design characteristics. As an illustration, cadmium telluride (CdTe) photovoltaic cell manufacturing is unique to MgCl_2_ treatment, which is used at the materials level to enhance grain structure and device performance. The process is not dependent on FPV structural design configuration and aquatic deployment criteria, and does not form a general consideration to floating photovoltaic system design. In this light, mention of MgCl_2_ is limited to the discussion of CdTe photovoltaic construction and is not discussed as a feature that defines FPV technologies.

### 1.3 Research Objectives and Review Scope

The primary aim of this review is to offer readers a thorough and systematic knowledge on the FPV technologies, their classification, engineering design methodologies, performance aspects, and their interactions with the aquatic environments. The integration of the various studies by the technological, environmental, and regulatory domains is urgently needed as FPV systems increasingly become a familiar and efficient, and land‐saving alternative to traditional solar PV. The review fills this gap by summarizing the literature of both peer‐reviewed and real‐life deployments.

Precisely, the purpose of the study is to:

- Classify various FPV system types and design approaches based on platform structure, mooring techniques, and energy generation characteristics;
- Highlight key engineering considerations affecting FPV performance, such as anchoring, water surface dynamics, cooling effects, structural stability, and auxiliary system;
- Examine the environmental consequences of FPV deployment on aquatic ecosystems, including impacts on water temperature, dissolved oxygen levels, biodiversity, and water quality;
- Conduct a life cycle and sustainability analysis comparing FPV systems with land‐based alternatives;
- Analyze the global Deployment of Floating Photovoltaic Systems;
- Present performance modeling and forecasting tools used for energy yield and ecological simulation;
- Identify existing policy frameworks and regulatory gaps that influence FPV adoption and long‐term viability.

The scope of the review is intentionally broad yet well‐defined: it includes inland and artificial water bodies (lakes, reservoirs, dams, ponds) and incorporates studies from 2000 to the present, with a strong focus on the post‐2010 commercialization boom. Hybrid fPV systems that combine hydro, wind, or aquaculture demonstrate the increasing adaptability of water surfaces. The review's goal is to provide essential tools for researchers, developers, engineers, and environmental policymakers to deal with the challenges of sustainable FPV deployment by finding a balance between environmental responsibility and technological innovation.

## 2 Historical Development of FPV Systems

A non‐conventional mechanism of generating solar energy is FPV, or floatovoltaics, which is the installation of solar panels on the surface of bodies of water such as lakes, reservoirs, and ponds. These systems enjoy a variety of advantages over traditional terrestrial solar, including reduced land requirements, higher efficiencies due to cooling by water, and the potential of accessing usually ineffective water surfaces [55]. The development of the FPV systems through history is divided into the following sections.

### 2.1 Inception and Initial Thoughts (Late 20th Century to Early 2000s)

The concept of floating Solar Panels emerged when scientists and engineers began to consider utilizing solar energy in locations where there is scarce land availability in the late 20th century. At this point, the materials and the technology that would then be required to produce reliable floating PV systems had not yet been developed [56]. Initial theoretical work focused on the possibility of installing solar panels on water bodies in order to reduce competition on land, particularly in areas with little land or having large populations to serve. A small number of experimental prototypes have been initiated in the early 2000s to attach solar panels to floating platforms in lakes or reservoirs [57]. These early attempts at floating solar laid the groundwork of the larger, more commercial floating solar projects that would follow in the years.

### 2.2 Commercialization and Early Pilot Projects (2007–2015)

A decisive milestone in the development of FPV technology was achieved in 2007 when the Institute of Advanced Industrial Science and Technology of Japan constructed the first floating solar construction in Aichi Prefecture [58]. Following the initial idea, a few pilot projects have been developed to consider the feasibility of FPV technology. In 2008, the Far Niente Winery in Oakville, California, built 994 such photovoltaic solar panels totaling 175 kW using 130 pontoons on the irrigation pond in the winery [59]. This installation demonstrated the practical applicability of the FPV systems in the business. ​The initial trade in plants of any organization is a 1 MW scale setup that was established in 2010 at the Kasai City Reservoir in Japan [60]. This was one large step in commercializing floating solar technology, and it was an improvement of what had been tried previously in size.

### 2.3 Growth and Expansion (2016–2020)

As the technology improved, FPV systems increased in size, performance, and also global acceptance. In 2016, China established the world's largest floating solar array, a 40 MW energy capacity plant at the Huainan Mine [61]. This experiment demonstrates the opportunities of floating solar when space is limited, as in the case in old mining areas. ​Some other countries, such as Vietnam, Thailand, Indonesia, and India, have been constructing floating solar farms and between 2017 and 2019, they have contributed to the spread of the technology across Asia [62]. The developed floating solar farms in Taiwan had an installed capacity of more than 100MW in the year 2018, strengthened by the Taiwan government's effort in renewable energy [63].

### 2.4 New Frontiers and Technological Developments (2020–Present)

**FIGURE 2:**
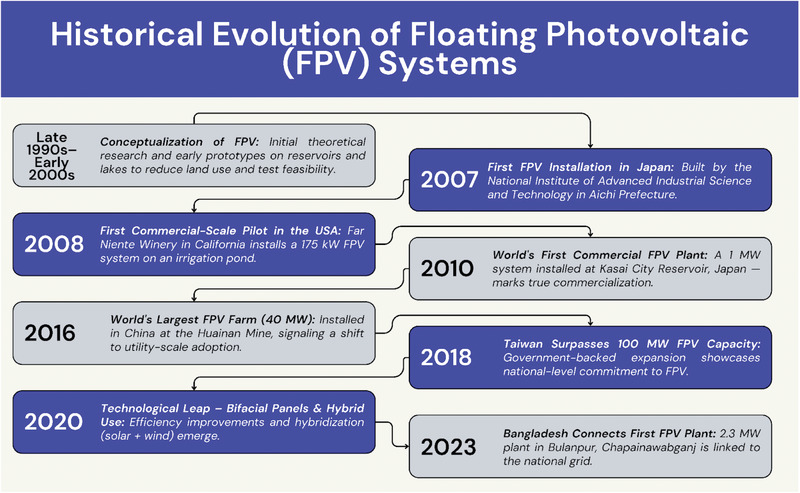
A timeline showing significant turning points in the creation and marketing of FPV technology, from inception to more recent developments. Copyright The Authors, 2025.

It is the needs of the market and the advances in technology that are driving innovation in the floating solar industry. In 2020, floating solar systems were much better in their design, durability, and performance. The development of bifacial solar panels that gather sunlight on both sides, front and back, began to increase the environmental output of the FPV systems [64]. There is a rise in the production of hydro‐floating platforms, which are offshore. ​Floating PV expanded again in 2021–2023, particularly where there is land‐constraint in solar farms. The initial installation of floating solar systems with their hybrid type solar wind power systems went to countries such as the UAE, Brazil, and Egypt [65]. Additionally, the initial floating solar power plant in Bangladesh is in Bulanpur of Chapainawabganj district, about 302 km away toward west of the capital, Dhaka. It is a 2.3 megawatts (MW) production capacity photovoltaic energy plant that was connected to the national grid in May 2023 [66]. The adoption of floating solar also became stronger in the US and Europe, with the projects installed in such countries as the Netherlands and the United States. A graphic overview of this development is shown in Figure 2, a timeline of the most significant milestones that have determined the development and the present‐day status of floating solar technology.

## 3 Methodology

This review uses a methodological literature approach of a structured, transparent, and reproducible approach to literature review in order to investigate the technological development of FPV systems, engineering design, environmental interactions, and sustainability issues. The methodology is oriented at a synthesis of the technical themes, as FPV research, due to its inter‐disciplinary character, involves a combination of PRISMA‐based principles of reviews with the synthesis of the research. This will provide a methodological rigor, and at the same time, it will be suitable for a technical and sustainability‐based review and not an experimental or statistical meta‐analysis.

### 3.1 Review Framework and Search Strategy

In this review, a structured literature review methodology was used, guided by PRISMA 2020, but modified to fit the technical and sustainability‐focused energy studies to achieve transparency and reproducibility in the literature selection. The aim was to critically compile and synthesize studies that covered the design of FPV systems, engineering performance, environmental dynamics, and sustainability impact.

A comprehensive literature search was conducted across major scientific databases, including Scopus (*n* = 142), Web of Science (*n* = 96), and ScienceDirect combined with IEEE Xplore (*n* = 74), yielding a total of 312 records from databases. Additional searches were conducted to supplement database searches, and minimize publication bias (websites, *n* = 34, organizational and institutional reports, *n* = 12, and citation searching, *n* = 28). Overall, there were 386 records that were initially identified.

The search queries were a combination of keywords that related to FPV technologies and applications, such as floating photovoltaic, floating solar, FPV systems, pontoon‐based FPV, flexible membrane FPV, and submerged photovoltaic, and terms that touched on the engineering design, environmental effects, life‐cycle sustainability, and considerations during deployment. The date range included 2000 to 2025, and those studies that were done after 2010 were prioritized to represent large‐scale deployment and recent sustainability measurements.

### 3.2 Study Selection

All the retrieved records were combined and filtered after identification to remove duplicates and apparent irrelevant material. The number of records that were deleted because of duplication was 74, and 22 records were deleted because of other reasons, such as incomplete information about the bibliography or non‐technical content. No records were deleted by automated screening tools.

Following these initial exclusions, there were 290 records left and were screened to title and abstract. In this step, 156 entries were filtered out because they were not relevant enough to FPV systems or because they were too technical. The rest of the 134 reports were requested to be retrieved in full‐text, where 11 were not available, and 123 reports were sent to complete the eligibility evaluation.

Peer‐reviewed papers were filtered out as they were not sufficiently technical, only dealt with non‐relevant uses of photovoltaics, or made conjectural assertions without analytical or empirical evidence. The redundant records and the opinion pieces that were not peer‐reviewed were also eliminated. This selection approach ensured that the resulting body of literature had technical rigor and practical relevance, and was consistent with the engineering focus of the review.

### 3.3 Screening and Eligibility Procedures

Full‐text eligibility analysis focused on the technical quality, relevance, and contribution of each of the studies to the understanding of the FPV system. A total of 123 reports were considered, and 73 reports were discarded because there were preclinical eligibility issues. Specifically, 31 articles were dropped due to lack of relevance to the FPV systems, 24 articles lacked sufficient technical or environmental analysis, and 18 reports were identified as duplicates or superseded project reports.

**FIGURE 3:**
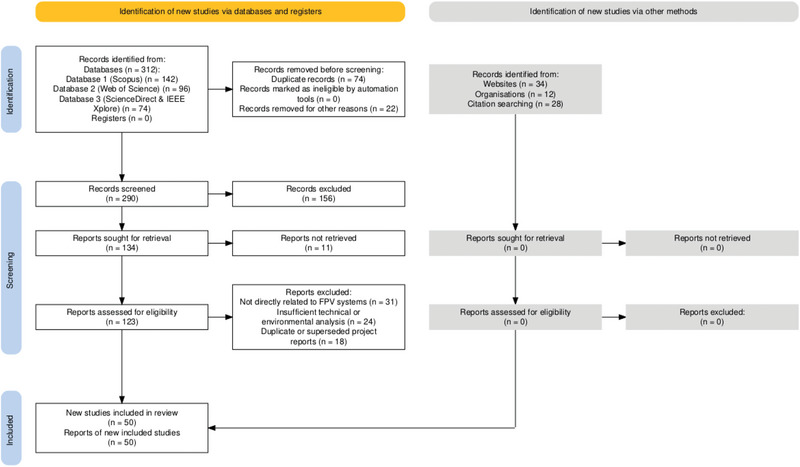
PRISMA 2020 flow diagram illustrating the identification, screening, eligibility assessment, and final selection of studies included in this review. Generated using a PRISMA diagram tool [67]. Copyright The Authors, 2025.

After this screening and eligibility, 50 papers were retained to undergo a qualitative and conceptual synthesis, as it met all the inclusion criteria. These researches were the foundational analysis of the review, involving the configurations of FPV systems, engineering performance, environmental effects, life‐cycle, and deployment experience. Figure 3 shows the entire process of identification, screening, and inclusion, and we employed a diagram generator [67]. The general identification, screening, eligibility, and inclusion processes are listed in a PRISMA 2020 flow, which gives a clear idea of the literature selection process.

### 3.4 Integration of the Conceptual Framework

In order to fill the gap of the fractured handling of FPV impacts and performance typically found in the current literature, the review adopts a synthesized conceptual framework that connects technical design decisions with environmental processes and sustainability outcomes. Instead of analyzing the FPV components separately, the framework brings into focus the interactions among the drivers of adoption, engineering design variables, aquatic ecosystems reactions, and the long‐term sustainability indicators.

**FIGURE 4:**
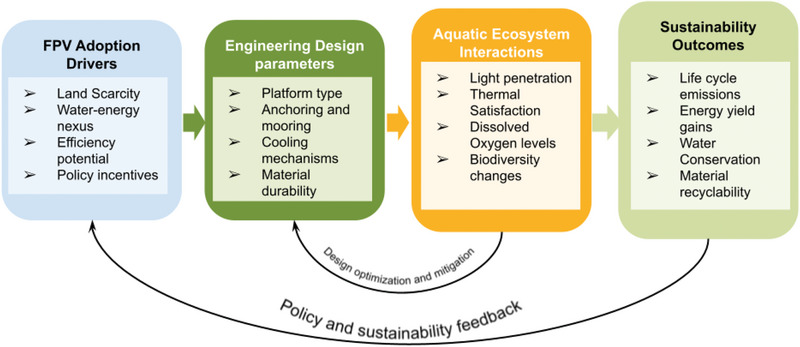
Conceptual framework illustrating the relationships between FPV adoption drivers, engineering design parameters, aquatic ecosystem interactions, and sustainability outcomes. Copyright The Authors, 2025.

These design choices, including the type of platform to use, anchoring methods, mooring methods, cooling methods, and the choice of material, directly influence system performance and interact with the environment, such as light penetration, water temperature, dissolved oxygen dynamics, and biodiversity changes. The framework also links these interactions to sustainability outcomes, which include life cycle emissions, energy payback time, water conservation, and material recyclability. The conceptual framework guiding this synthesis is illustrated in Figure 4.

## 4 Classifications and Components of FPV

**FIGURE 5:**
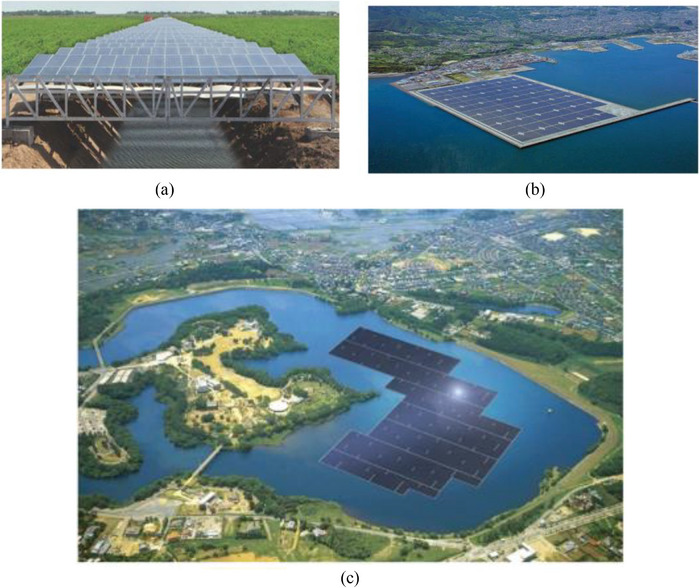
Different types of solar pv installations: (a) canal‐top solar pv system utilized over water conveyance channels is adapted from Ref. [70] under the terms of the Creative Commons CC BY license, (b) offshore solar pv plant deployed in marine environments, (c) floating solar pv system installed on inland water bodies which schematic representations based on publicly reported floating solar deployments described in Refs. [71, 72].

The growing global demand for clean energy has driven solar PV technology to expand beyond traditional land‐based installations into innovative applications that optimize space utilization and improve efficiency. These include setting up the PV systems on the inland water bodies, maritime habitats, and over water channels. These procedures are effective in enhancing performance through water evaporation reduction, minimization of land‐use conflicts, and enhancing cooling effects to solar panels, besides generating sustainable electricity. Figure 5 illustrates an innovative solar PV installation that aims at overcoming the problem of land scarcity and enhancing efficiency. Inland waters Floating systems use the cooling effect of water to improve performance, marine regions that have natural cooling advantages, and canal‐top systems save land and reduce evaporation. Together, the above measures demonstrate that the floating and water‐based PV systems can be effective renewable energy sources in land‐strained regions. The FPV systems have an average efficiency that is 11% higher than their ground‐mounted counterparts [68, 69]. Overall, the combination of improved energy yield, reduced land dependency, and environmental adaptability makes FPV an increasingly attractive option.

### 4.1 Classification Based on Design

#### 4.1.1 Pontoon‐Based FPV

The most popular and widely implemented type of FPV technology is based on pontoons, which is related to their modularity and scalability, as well as reliability. In such systems, pontoon buoyant floats are used, which give adequate stability to hold the solar modules and other equipment on top of the water. Pontoons are commonly rotationally molded using medium‐ or high‐density polyethylene (MDPE/HDPE), which can be more cost‐effective than using steel in the long term due to UV resistance, resistance to corrosion, and biofouling [73, 74]. In addition to buoyancy, most pontoons can have grooves and channels incorporated to manage cables, drainage, safety and easy maintenance access, to aid system integration and operation.

**FIGURE 6:**
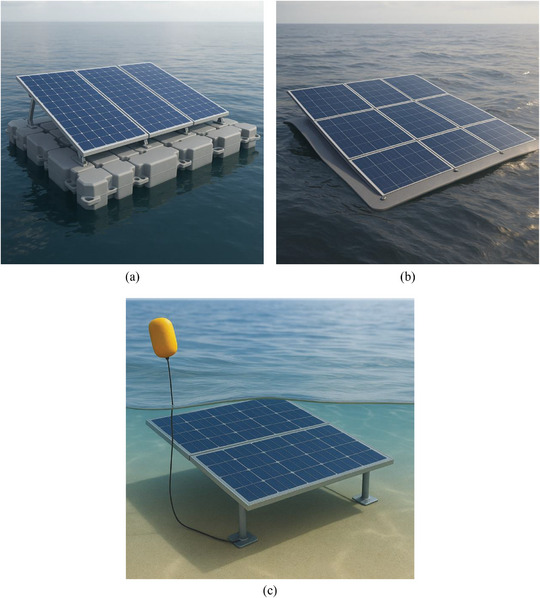
Classification of FPV systems based on design approaches: (a) Pontoon‐based FPV, utilizing modular floating pontoons to provide buoyant support and structural integrity; (b) Flexible membrane FPV, employing a thin‐film flexible array that conforms to water surface dynamics for improved adaptability and wave resistance; (c) Submerged FPV, representing experimental or pilot‐scale concepts in which photovoltaic modules are partially or fully immersed for research on thermal and optical interactions, rather than commercial power generation. Copyright The Authors, 2025.

Pontoon‐based FPV systems have become the most widely implemented due to their scalability, modularity, and proven reliability. They use durable HDPE or MDPE pontoons that resist corrosion, UV degradation, and biofouling while also integrating features like cable channels and walkways to aid maintenance. In comparison to ground‐mounted systems, the floating platform's solar modules are attached at optimum tilts, and their close proximity to water allows for natural cooling, improving energy conversion efficiency. Even though they increase installation costs, anchoring and mooring are necessary to avoid drift or displacement brought on by wind, waves, or changing water levels, ensuring dependable and safe operation. The design's global flexibility is highlighted by its deployment in reservoirs, hydroelectric dams, and artificial lakes around the world. Regionally, large‐scale FPV deployment has been led mostly by Asia, while European activity has concentrated on pilot‐scale projects, hybrid FPV–hydropower systems, and policy‐supported demonstrations. Despite these advantages, pontoon systems have drawbacks due to their need for strong anchoring, potential impact on aquatic ecosystems through decreased solar penetration, and requirement to withstand environmental pressures. Large arrays can also be logistically difficult to move and assemble, especially in isolated locations. Furthermore, logistical challenges may arise while transporting and installing big arrays, especially in remote areas. However, as shown in Figure 6a, pontoon‐based FPV is the basis for current and future floating solar systems due to its scalability, adaptability, and dependability.

#### 4.1.2 Flexible Membrane FPV

The flexible membrane FPV systems are made of thin‐path solar modules, which have been mounted on the flat and rigid platforms standing directly on the water surface. These systems are constructed with polymeric or composite membranes as opposed to rigid pontoons, in which the membrane can flex according to the wave motions to cause less mechanical strain on the membrane and damage to the modules may occur. Thin‐film photovoltaics such as amorphous silicon, CdTe, or CIGS are usually utilized, and have proven to be more resistant to high temperatures, partial shade, and diffuse light than crystalline modules. The fact that the membranes are very near the water surface uses the surface tension and reduces the lifting caused by the water flow, in addition to ensuring that the modules are aligned even in very fluid waters.

FPV systems based on flexible membranes represent lightweight and flexible systems that are especially used in irrigation canals, industrial ponds, and other water bodies where rigid pontoon systems do not make sense. Their thin‐film solar modules, like CdTe or CIGS, can be used to work under high temperatures, partial shading, and diffuse light conditions, and their proximity of the water surface offers them a great cooling mechanism and optical performance. Such a layout makes installation a simple task, minimizes structural needs, and leads to higher power output, in studies it has been reported that this layout may increase the yearly output by up to 5% over ground‐mounted PV installations [75, 76]. However, in spite of these benefits, there are a number of challenges associated with flexible membrane systems that prevent the widespread adoption. The fact that polymeric membranes are vulnerable to UV degradation, punctures caused by floating debris, and biofouling, and that their mechanical strength is relatively low, restricts their application in more challenging settings. Repairs or replacement might prove to be frequent and make it more difficult to maintain and less economically viable. Nevertheless, the technology remains promising and innovative, with the potential to significantly expand the application of floating solar power beyond conventional sites, as illustrated in Figure 6b.

#### 4.1.3 Submerged Floating PV

Submerged FPV systems are a form of experimental set‐ups where photovoltaic panels are partially or wholly submerged beneath the water surface, and the main aim of the structure is to study thermal control and interactions of photovoltaics and water instead of facilitating direct commercial implementation. Direct exposure to water in such systems can lower the operating temperatures in modules, thus alleviating the performance losses due to thermal effects caused by the temperature in controlled environments. The experimental research by Tina et al. and Lanzafame et al. has demonstrated that shallow submergence of photovoltaic modules, usually at depths in the range of several millimeters to centimeters, can result in short‐term performances and local efficiency increment in laboratory or pilot scale applications [77, 78]. However, the performance of submerged FPV systems is strongly constrained by optical limitations associated with water immersion. Light attenuation, spectral filtering, surface reflections, and water turbidity substantially reduce the effective irradiance reaching submerged modules, particularly as immersion depth increases. Consequently, the efficiency gains have been reported to be very sensitive to the clarity of the water, the depth of immersion, and the control over the experiment, and the gains can not be directly applied to large field conditions. The SCINTEC submerged PV design is just one example of innovative ideas operating on very shallow immersion lengths (around 02 mm), but aiming to strike a balance between thermal advantages and reduced optical losses instead of allowing solar energy to be generated underwater [78].

In addition to optical factors, submerged FPV systems also have several practical issues that restrict scalability. These comprise a higher level of vulnerability to biofouling, sedimentation, and algal growth that may lead to low performance and an increment in maintenance needs. In addition, there should be a proper anchoring and positioning mechanism so as to have a fixed level of immersion where fluctuation of water levels and moving water systems exist, and this poses additional engineering challenges. In this review, submerged FPV is discussed to provide insight into ongoing experimental efforts aimed at improving photovoltaic thermal management and understanding PV–water interactions, while remaining clearly distinct from the pontoon‐based and flexible membrane systems that currently dominate practical FPV deployment, as illustrated in Figure 6c.

Among the three main structural designs of floating photovoltaic systems (pontoon‐based, flexible membrane‐based, and submerged type), the pontoon‐based has become the most commonly used and technically proficient. A number of large‐scale and pilot projects across the world reinforce the use of the three major structural designs of the floating photovoltaic system. Table 2 focuses on the key large‐scale and pilot FPV applications.

**TABLE 2:** Notable FPV projects Worldwide based on structural design.

FPV type	Project/System name	Country	Capacity	Refs.
Pontoon‐based FPV	Duriangkang FPV project	Indonesia	2200 MW	[42, 44]
	Saemangeum FPV	South Korea	2100 MW	[42, 44]
	Wenzhou Taihan FPV	China	550 MW	[42, 44]
	Ramagundam FPV (NTPC)	India	100 MW	[42, 44]
	Cirata reservoir FPV	Indonesia	145 MW	[79, 80, 81]
Flexible membrane FPV	Ocean Sun BOOST	Spain	275 kWp	[82]
	Soneva secret FPV	Maldives	2 MW	[83]
	Ocean Sun–Keppel membrane pilot	Jurong Island, Singapore	Pilot (∼1.5 MW)	[84]
Submerged FPV	Hide & shine pilot	Netherlands	Pilot (∼100 kW)	[85]

As shown in Table 2, pontoon‐based FPV systems clearly dominate global deployment, with multiple utility‐scale installations exceeding hundreds of megawatts, particularly in Asia. Flexible membrane FPV systems are being developed mainly by pilot demonstrations and preliminary commercial demonstrations in particular settings (offshore, in the presence of waves), where lightweight and flexible structures can be beneficial. By contrast, submerged FPV designs are still limited to experimental and pilot‐scale applications and are still a research‐focused idea and not a commercially developed technology. This allocation shows a very clear technological hierarchy in the development of FPV, with the most established and scalable solution the pontoon‐based systems, the most in the transitional niche is the flexible membrane systems, and the submerged FPV is still a matter of research and development.

### 4.2 FPV System Components

#### 4.2.1 PV Modules and Floating Structures

PV modules with FPV systems typically offer a mixture of efficiency and low‐cost as well as reliability by utilizing normal crystalline silicon solar cells. PV modules placed on water surfaces, however, require unique considerations, especially with regard to moisture absorption and corrosion resistance [86]. Modules must be particularly made to survive extended exposure to salt mist for use on saline water bodies. Stainless steel or polymer‐based frames are corrosion‐resistant substitutes for standard aluminium frames and mounts because of their propensity to corrode [87].

Pontoons or floats are the main types of floating structures that support these PV modules. Pontoons are large floating platform and have sufficient buoyancy to hold the weight of PV modules and associated equipment. These pontoons are normally constructed with high‐density polyethylene (HDPE), which is appreciated because it gives a great strength‐to‐weight ratio, is corrosion‐resistant, UV rays, and also recyclable [88]. Alternatively, it is also possible to use glass fibre reinforced plastic (GRP) because of the same beneficial nature. Floats are steady and scalable platforms of floating that consist of many multiplexed hollow components made of plastic that create large floating arrays [89].

#### 4.2.2 Anchoring and Mooring Systems

The most essential systems are anchoring and mooring, which would stabilize and anchor the FPV settings. They secure the floating arrays against the wind and the currents, as well as other environmental aspects. Depending on the site's unique environmental characteristics, the mooring system usually consists of anchors, cables, chains, and connection points like buoys or bollards [90]. Mooring systems often use wire rope slings or nylon ropes attached to bollards on the shore for installations on shallow or generally calm bodies of water. On the other hand, complex anchoring methods, such as concrete anchors or submerged anchor blocks, are needed for installations on deeper or more turbulent water bodies [91].

These systems are location‐specific in that they consider hydrodynamic conditions (waves, currents), wind load, water depth, seabed/lakebed composition, and environmental limitations. The anchors must be capable of bearing dynamic loads and must be capable of resisting corrosion and fatigue, and therefore, a regular inspection and maintenance routine is necessary to ensure the anchors are useful in the long term.

#### 4.2.3 Inverters and Monitoring Units

The inverters are another necessary electrical component of FPV systems, and they convert direct current (DC) electricity produced by the PV modules into alternating current (AC) to be integrated at the grid level or to be utilized locally. In FPV, inverters of this type are usually land‐based, such that they do not have direct interaction with moisture and corrosion [92]. The significance of such inverters being efficient and reliable is very high because the dimensions influence the performance and generation of energy in the system.

**FIGURE 7:**
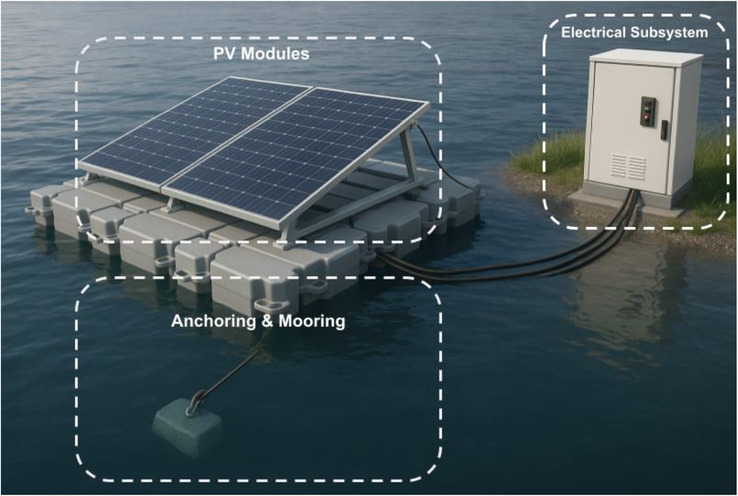
A detailed 3D representation of a standard FPV system that highlights each of its component parts. Copyright The Authors, 2025.

FPV system monitoring units play a crucial role as they continuously supplement us about how the systems are working, their health status, and the environmental status. These systems have solar irradiance, temperature, voltage, current, and moisture ingress sensors. The monitoring of the data collected is continually reported to the central monitoring device through wireless or physical transmission service [93]. This kind of monitoring enables predictive maintenance, earlier faults in detection, and optimization of performance. High‐end monitoring systems involve the use of digital twins and AI‐powered predictive analytics to increase the reliability of operations and maximize energy production of FPV installations [94]. Figure 7 highlights FPV modules, HDPE pontoons, anchoring and mooring systems, sturdy waterproof wires, and onshore inverter units.

**FIGURE 8:**
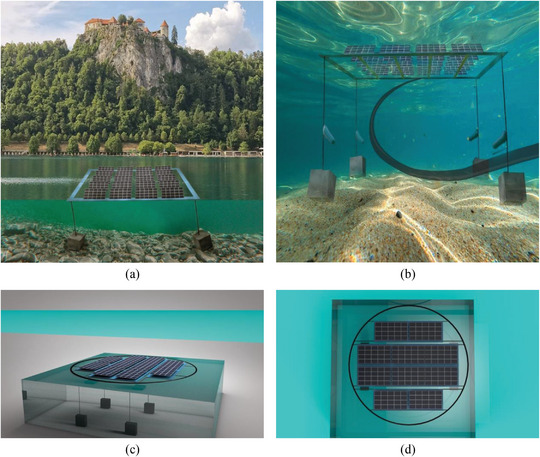
3D visualization of FPV system configurations and structural deployment. (a) Realistic shoreline‐side installation illustrating surface‐level mounting and mooring. (b) Underwater perspective showing anchoring and cable routing for submerged system support. (c) Oblique 3D simulation of a square FPV array with mooring blocks and structural distribution in semi‐transparent water. (d) Top‐down simulation illustrating panel layout symmetry and circular mooring arrangement on a still water surface. Copyright The Authors, 2025.

This section illustrates 3D representations of several design concepts to obtain a better idea of the structure of FPV systems and how they are shaped spatially. They provide layout, buoyancy systems, panel position, and insertion into the water bodies based on these models, indicating the perspective on functional and visual points of view on FPV construction. This area helps the technical discussion by coming up with a physical representation of how floating solar systems are implemented in real‐life contexts. There is an FPV system 3D visualization demonstrated in Figure 8.

## 5 Engineering Strategies

### 5.1 Site Selection and Environmental Resilience

In order to ensure platform stability and system longevity, the placement of FPV installations is a complicated procedure that is required for both hydrological and underwater assessments. The typical places of ideal FPV include smooth inland water bodies like hydroelectric dams, reservoirs, and drinking water basins, which give consistent conditions on which the installations will be located [95, 96]. The need to evaluate and select the best sites through a multi‐criteria decision‐making framework that relies on Fuzzy Analytical Hierarchy Process (FAHP) and Geographic Information Systems (GIS) techniques can result in the effective and precise determination of the best sites on the basis of technical, economic, and ecological criteria [97].

Sea resistance to waves, wind, and corrosion control measures are all part of environmental resilience considerations. FPV installations located offshore or on land require robust platforms and have to endure most adverse conditions, which requires a platform design developed to meet the requirements of standards such as API 2A and Japanese Class NK [98, 99, 100]. These specifications center on designing FPV structures that can endure extreme weather conditions, ensuring minimal disruptions and preserving the long‐term viability of operations.

### 5.2 Mooring and Structural Hydrodynamics

The FPV systems are not stable enough, in that the floating system will be susceptible to dynamic water forces, tides, and wind. The hydrodynamic and structural interactions involved in such interactions necessitate the modeling of such interactions in an attempt to make the platforms stable and dependable.

Finite Element Modeling (FEM) has been used to assess the thermal and mechanical behavior of FPV structures under real‐world conditions, and it has been observed that the structure in question performs well, both in wind, waves, and temperature gradient conditions [101, 102]. Although FEM aids in determining the mechanical integrity of modular FPV platforms, gigawatt‐scale capability can be realised by deploying large FPV arrays in more than one water body. It has been proposed that even a minor portion, say 5%–10% of existing reservoir surfaces, would be able to cover gigawatt‐scale solar power, depending on the geography of the area and access to water [103].

The mooring configurations can also be improved with optimization models that measure cable tension, anchor strength, and floatation responses to various environmental conditions [104]. These simulations help engineers design FPV arrays that can resist cyclones, wave surges, and strong winds, lowering the risk of system failure. Another emerging area is the development of hybrid mooring systems. These systems combine standard anchoring with elastic or semi‐elastic components that absorb very high dynamic stresses. This approach extends the lifespan of cables and connectors and reduces stress on anchor points.

Furthermore, long‐term projections of FPV constructions' performance under climate change scenarios such as rising water levels, shifting wind patterns, and an increase in the frequency of extreme weather events are now feasible by fusing hydrodynamic modeling with climate resilience research. In large‐scale FPV projects, these predictive models offer useful information for long‐term risk assessment and planning.

### 5.3 Energy Efficiency and System Optimization

FPV systems need to be power efficient, and to a large extent, thermal regulation and efficient irradiance capture is a significant factors. In comparison to terrestrial photovoltaic systems, being close to bodies of water can reduce the modules' working temperatures through passive cooling, which can minimize performance losses brought on by thermogenic effects. Studies by Saxena et al. [105] and Mamatha & Kulkarni [106] indicate that such cooling effects can lead to moderate efficiency improvements under hot climatic conditions, particularly when FPV systems are compared with ground‐mounted PV installations operating under similar irradiance levels. The appropriate structural support of in place items such as glass‐fiber‐reinforced plastic (GFRP), among other materials with light weights, contributes significantly to the overall effects of the floating structures and placement of panels where the loads are located [107]. Besides these high‐level design principles, the type of floating structures and the positioning of the panels significantly influence the overall performance outcomes. The pontoon‐type or surface‐floating photovoltaic systems have strong evaporative cooling and lower operating temperatures because panels are supported barely above the water on floating supports. However, biofouling and waves that cause mechanical stress may have an impact on them. Flexible membrane‐type FPV systems also offer strong cooling advantages and place solar panels on expansive sheets of floating material directly on the surface of the water. These systems frequently use lower module temperatures and more energy efficiently because of their proximity to the water. Nevertheless, these systems are more prone to wind, wave motions, and long‐term mechanical forces as the membrane is moving with the water body, which can affect the structural reliability. Submerged‐type FPV, in which the modules are largely or entirely immersed in water to achieve direct water cooling, but finds application in utility‐scale applications less often because anchoring and maintaining stability are more difficult on large reservoirs.

Tilt angle and panel elevation optimization are also necessary in order to maximize the FPV efficiency. In tropical or equatorial locations, panels positioned at modest tilt angles, typically between 5° and 10°, offer higher structural stability against wind loads and reduce shadowing between rows [108]. These designs may interfere with irradiance collecting at higher latitudes, when steeper tilt angles would be required to match sun patterns and increase energy efficiency. Although steeper angles might make a platform more vulnerable to wind‐induced stress and instability, they also help with natural cleaning by rain, which reduces dust collection. The panels' height above the water's surface also introduces performance trade‐offs: panels nearer the water's surface benefit most from cooling but are more susceptible to moisture and splashing, while panels higher up are more resilient and simpler to maintain but lose some of the cooling efficiency gains. According to research, the efficiency gains from water cooling progressively diminish with increasing distance from the surface; raised arrays provide superior long‐term reliability, while submerged or near‐surface systems typically achieve the largest improvements. By modifying tilt, spacing, and platform height in accordance with the climatic and hydrological parameters of the deployment site, many designs in practice use hybrid approaches that strike a balance between cooling‐induced efficiency and structural durability.

### 5.4 Auxiliary System for Performance Enhancement

FPV systems depend on auxiliary systems that support and improve their functionality in addition to their main energy generation components to guarantee peak performance and long operational lifespan. Even though they are not directly involved in power conversion, these auxiliary systems are essential to preserving the ideal environmental conditions surrounding the solar modules and ensuring long‐term efficiency. Systems for cooling and cleaning are among the most important because they assist in controlling module temperature and reduce performance losses brought on by surface contaminants [109]. The design and performance implications of various auxiliary systems in relation to FPV deployment are discussed in this section.

#### 5.4.1 Cooling System

In addition to the passive cooling benefits inherent to FPV deployment, auxiliary cooling systems have been investigated to support temperature regulation under high‐irradiance or site‐specific operating conditions. These systems are intended to complement, rather than replace, the natural cooling effects discussed in Section 4.3, and are primarily evaluated in experimental or pilot‐scale studies. According to modeling studies, Golroodbari and van Sark [110] calculated that, in comparison to an on‐shore PV farm, the PV cooling effects of a floating solar farm might lead to an average yearly output energy increase of 12% to 18%. Therefore, cooling solutions are crucial for PV facilities to withstand the impacts of heat rising due to sun radiation and keep the PV panel temperature within the appropriate working ranges. Certain cooling strategies that were first studied for onshore photovoltaic systems [111] have demonstrated encouraging outcomes and may serve as models for floating applications. Active cooling techniques are nevertheless essential for keeping solar modules within ideal temperature ranges, particularly in high‐irradiance environments, despite these passive advantages. Early cooling techniques created for PV systems on land have proven successful and are now being modified for FPV platforms. The availability of water in FPV systems makes water‐based cooling technologies particularly viable among them. One method that helps with temperature control and system efficiency is to spray water directly onto the PV panel surfaces. The water‐based cooling method involves spraying panel surfaces with water [112, 113]. A number of economic feasibility analyses were performed by de Oliveira Busson et al. [114] and Micheli [113]. Micheli and de Oliveira Busson have provided evidence for the applicability of these approaches through experimental research and economic feasibility assessments. The application of water spray over panel surfaces is a component of the water‐based cooling approach.

In order to lower temperatures and boost system efficiency, Sheikh et al. [115] presented a lightweight, minimally intrusive forced water cooling system design in another study. Beneath the PV panels, a water circulation system was installed. This system uses water from below to pump up water, remove heat, and return it. Kjeldstad et al. [116] assessed the effects of water cooling on a floating membrane‐type FPV system that has PV modules arranged horizontally and that make thermal contact with water. Economic assessments by Tina et al. [117] have demonstrated that implementing cooling techniques into FPV systems can greatly boost energy yield while preserving cost‐effectiveness. Recent comparison studies are still being conducted to examine the performance improvements that come from different cooling solutions for floating PV installations [118, 119].

#### 5.4.2 Panel Cleaning System

Maintaining clean photovoltaic panel surfaces is essential to ensure consistent energy output and system reliability. The presence of dust, bird droppings, and other airborne particles collectively referred to as soiling can significantly reduce the amount of sunlight reaching the solar cells, leading to substantial performance degradation [120]. It is essential to clean the solar panels as needed because dust buildup can reduce the power output of PV panels by up to 15% every day [121]. According to a review by Deb et al. [122], FPV offers the convenience of using water‐based cleaning techniques, which are thought to be efficient and economical. Additional advantages of water‐based PV cell washing techniques include a reduction in operating temperature [123]. To prevent this, a tilting angle of the panels may be taken into consideration. If the water remains on the panel surfaces, it may alter the reflected light [122] and induce solar spectrum modification. Adding an automatic mechanical device to clean the panels is another option, but it might be the most expensive [124]. Panel cleaning systems primarily contribute to performance stability by minimizing soiling‐related losses and supporting long‐term operational reliability, rather than serving as a direct mechanism for efficiency enhancement.

### 5.5 Maintenance, Reliability, and Safety

The long‐term viability of FPV operations depends significantly on key maintenance, dependability, and safety standards. Regular visits, supported by automated and digital surveillance systems, would require early, proactive identification and resolution of any potential issue. Some common maintenance actions are regular checks, periodic cleaning of the surface, repair of damaged parts, and replacement of the part, which is important because of the challenges in the aquatic environment like biofouling and corrosion [125].

FPV system safety and reliability are partly associated with risk mitigation measures. The risks linked to contact with water, such as the possibility of electrical hazards and deterioration of equipment, are mitigated by electrical isolation, as well as extensive weatherproofing procedures. In particular, new waterproof cables and corrosion‐resistant cables, as well as secure electrical parts that can be developed and involved in the systems, increase the safety of systems considerably [126]. Regular and thorough care and strong risk management strategies already go a long way in increasing the FPV system reliability, performance, and functional persistence [127, 128, 129]. Key strategies and the factors affected in the FPV system are highlighted in Table 3.

**TABLE 3:** Comprehensive overview of engineering strategies for FPV systems.

Design strategy	Key factors	Considerations
Energy efficiency	Cooling effects	Utilize water's natural cooling to reduce panel temperatures, contributing to moderate performance improvements under favorable climatic conditions [105, 106].
	Optimal irradiance capture	Determine optimal tilt angles and panel layouts, reduce shading losses, and maximize sunlight exposure.
	Material selection	Employ robust, lightweight materials like Glass‐Fiber‐Reinforced Plastic (GFRP) to ensure durability and stability [107].
Site selection	Bathymetric & hydrological assessments	Evaluate water depth, reservoir bed composition, and water flow dynamics for platform stability.
	GIS‐based analysis	Use geographic information systems (GIS) combined with multi‐criteria decision making methods for accurate site assessment [97].
	Ecological and economic criteria	Balance environmental sensitivity and biodiversity impacts with economic feasibility, proximity to grid infrastructure, and overall cost‐effectiveness [95, 96].
Environmental resilience	Wave and wind resistance	Design FPV systems to endure significant wave actions and high wind conditions, applying standards like API 2A and Japanese Class NK [99, 100, 107].
	Corrosion mitigation	Apply corrosion‐resistant materials and protective coatings to withstand harsh aquatic environments, ensuring long‐term structural integrity.
Maintenance and reliability	Inspection frequency	Schedule regular and systematic inspections, supplemented by automated monitoring systems, to proactively manage system integrity and performance.
	Biofouling and corrosion	Implement comprehensive strategies for periodic cleaning and component replacements, addressing biofouling and corrosion challenges specific to aquatic environments [130].
	Digital monitoring systems	Integrate advanced digital monitoring systems for real‐time operational tracking, performance optimization, and predictive maintenance.
Safety	Electrical isolation	Ensure strict electrical isolation practices to minimize risks associated with water exposure, reducing potential for electrical faults and human hazards [126].
	Weatherproofing and waterproofing	Use robust, waterproof, and weather‐resistant materials and designs to protect electrical components and maintain operational safety.
	Secure electrical components	Utilize IP‐rated waterproof cables, junction boxes, and secure electrical fittings, reducing the risks associated with aquatic exposure [126, 127, 128, 129].

The long‐term viability of floating photovoltaic systems is governed by system‐level failure modes, regulatory constraints, and operational interactions with existing water infrastructure, beyond component‐level performance considerations. Prolonged exposure to ultraviolet radiation, wave‐induced cyclic loading, and temperature fluctuations can lead to polymer fatigue and mechanical degradation of floating structures and mooring components over time. High‐humidity and water‐adjacent operating conditions also elevate the risk of electrical insulation failure, corrosion, and leakage currents, necessitating stringent waterproofing, grounding, and safety standards [92]. In parallel, biofouling and sediment accumulation can accelerate material degradation, increase maintenance frequency, and reduce system reliability if not properly managed. From a governance perspective, FPV deployment is often constrained by water‐body ownership, multi‐use permitting frameworks, and regulatory oversight related to drinking water supply, irrigation, and ecosystem protection [39]. In hydropower reservoirs, additional operational challenges arise from FPV–hydropower interactions, including fluctuating water levels, access requirements, safety clearances, and potential impacts on dam operation and maintenance schedules.

## 6 Environmental Consequences and Ecosystem Interactions

### 6.1 Aquatic Light Penetration and Vegetation Impacts

FPV systems have a drastic effect on aquatic life, this is because they alter the availability of light by significant values in the water body. Large areas of the water surface are normally covered with FPV installations that can vary between 30% to 50% and even more, resulting in a substantial decrease in Photosynthetically Active Radiation (PAR) [131]. Research has shown that PAR under FPV panels can drop by up to 95%, which severely inhibits the photosynthetic activity of aquatic plants submerged in water. For instance, studies in the Netherlands found that the amount of submerged vegetation under FPV installations had tripled, underscoring the possibility of significant changes in plant biomass and ecosystem structure [132]. This broad shading is therefore likely to reorganize fish and other aquatic food webs, alter fish and other living wildlife habitats, and limit proper control of FPV coverages. Strict ecological control is required to minimise disturbances and ensure the FPV systems operate sustainably, in addition to covering only eco‐safe levels [133].

### 6.2 Changes in Water Temperature and Stratification

Installation of FPV impacts water systems in the sense that it alters the dynamics of Stratification and temperature. FPV modules over water reduce the temperature fluctuations in water and usually cause surface temperatures to lower considerably [134]. As an example, multi‐year observational studies of French lakes noted that average surface water temperatures declined by around 1.2°C, and the biggest variations by up to 3°C during the heat waves. Also, simulated models in the Netherlands showed a maximum of 8°C in reduced surface and an enhancement of 200 days in duration of thermal stratification [135]. These shifts have the potential of stabilizing water columns, which decreases mixing and may shift nutrient cycling and aquatic ecosystems.In order to minimise potential impacts on aquatic life and ecological processes, careful ecological consideration and adaptive guidance are essential [136]. For instance, changes in mixing energies of about 22% have been observed [137].

### 6.3 Biodiversity and Aquatic Life

FPV installations influence aquatic ecosystems primarily through biological and ecological pathways, beyond physical changes in light availability and water temperature. These impacts are reflected in changes to fish behavior, breeding patterns, habitat availability, and broader biodiversity dynamics, which are often mediated by altered food‐web interactions and habitat structure rather than direct physicochemical changes alone. As the studies have shown, FPV systems can potentially disorient major socio‐ecological practices to which local communities rely, including fishing and recreation [138]. According to other research, FPV installations may have an impact on a broader sustainable development goal and change important natural processes like carbon capture and storage and water purification [139].

FPV causes complex environmental changes in aquatic habitats by altering physical, chemical, and biological processes [140]. Additionally, FPV structural components and mooring lines present physical risks to aquatic life, such as breakage or a dangerous trap [138, 141].

Indirect consequences, such as disruptions in nutrition cycles, are as common but harder to predict. FPV panels' shading reduces the amount of sunlight that reaches the water, harming the photosynthesis of aquatic plants and algae, which form the base of aquatic food webs. Low primary production and variations in thermal structure may have a major effect on the distribution and survival of aquatic species, particularly those that are temperature‐sensitive. The exact impact and its consequences are shown in Table 4.

**TABLE 4:** Comprehensive overview of FPV impacts on biodiversity and aquatic life.

Impact category	Specific impact	Description and ecological consequences	Refs.
Direct physical	Entrapment and damage	Risks from mooring lines and FPV structural components leading to injuries or fatalities of aquatic organisms.	[117, 138]
	Shading	Reduced sunlight penetration impacts aquatic plant photosynthesis, leading to lower primary production and disruption of food webs.	[142, 143]
Thermal and chemical	Temperature and oxygen levels	Shading‐induced modifications in water temperature and decreased dissolved oxygen, potentially creating anoxic conditions harmful to species.	[139, 143]
	Nutrient cycles	Alterations in nitrate nitrogen and phosphorus concentrations affect phytoplankton growth and overall water quality.	[139]
Socio‐ecological	Fishing and recreational activities	Changes in fish behavior and populations are affecting local fishing economies and recreational activities.	[138]
Ecosystem services	Water purification and carbon storage	Potential disruptions to essential ecosystem services, such as water filtration and carbon sequestration capacities of aquatic ecosystems.	[139]
Biodiversity	Habitat alteration	Modification of habitats due to FPV installations potentially leads to loss of biodiversity, especially in ecologically rich inland waters.	[142]
	Invasive species risks	Increased risk of invasive species colonization facilitated by structural alterations and habitat changes associated with FPV deployments.	[117, 142]

In addition, a chemical composition shift of the water bodies, like a change in nitrate nitrogen or available phosphorus, has consequences for phytoplankton growth and the overall ecosystem conditions. It is therefore important that these various effects be evaluated with great caution and care in order to ensure that minimal ecological disturbances occur.

### 6.4 Water Quality and Biochemical Parameters

In addition to the physical and thermal changes discussed earlier, FPV systems influence water quality through measurable biochemical indicators such as dissolved oxygen (DO), pH, turbidity, and nutrient concentrations. FPV installations research has demonstrated that the levels of dissolved oxygen have reduced significantly, with ranges of about a reduction of 1.1 to 1.7 mg/L being observed under FPV panels in the Netherlands [144]. Likewise, in the experiment, the DO declined by 4.14 to 3.5 mg/L or more with large FPV coverage [145]. Also, minor changes in pH (approximately ±0.1–0.3 units), turbidity, and total organic carbon (approximately 15%) have been reported. The alterations may affect the living environment or the learning of the aquatic animals and the well‐being of these organisms [146].

In addition, the FPV installations unintentionally facilitate the growth of eutrophication and algal bloom incidence, especially in water bodies that are rich in nutrients. As an example, model experiments with reservoirs in Singapore showed that chlorophyll‐a, nitrogen, and phosphorus revealed a significant increase of the series by 30%, 10%, and 30%, respectively, and in FPV installations, there were serious decreases in DO (up to 50%). Therefore, there must be constant patrol and predictive ecological modeling, which will help curb any adverse effects and maintain the ecological balance that will enable the deployment of the FPV systems in an environmentally responsible manner [147].

### 6.5 Environmental Mitigation Approaches

In order to lessen the environmental impact associated with floating photovoltaic systems, such mitigating techniques would be necessary. The use of mitigation techniques, such as precision of the coverage ratio, (distance between the modules, and anchoring locations are based on maximization of efficiency generation and ecological sustainability. A reduction in the photosynthetic activity of aquatic plants, a change in thermostatification, a drop in the concentration of dissolved oxygen, and changes to the environment can all be prevented or greatly reduced with careful management of these factors [148]. The goal of these mitigation efforts is to find a balance between boosting energy efficiency and causing the least amount of disruption to the ecosystem. Table 5 shows the comprehensive mitigation strategies that helped to address the identified ecological issues (Table 5).

**TABLE 5:** Strategies for addressing environmental issues in FPV systems.

Parameter	Mitigation approach	Ecological concerns mitigated	Benefits	Implementation difficulty	Refs.
Coverage ratio	Limit FPV coverage to less than 50%	Reduces shading, maintains adequate sunlight penetration, biodiversity, and oxygen dynamics	Enhances ecosystem balance	Moderate	[142, 143]
Module spacing	Increase spacing between FPV modules	Enhances sunlight penetration, improves water mixing, reduces thermal stratification, and maintains dissolved oxygen levels	Promotes healthier aquatic habitats	Low	[143]
Anchoring positions	Strategic placement away from sensitive habitats	Minimizes physical damage to benthic ecosystems, reduces entrapment and habitat alteration risks	Protects sensitive ecological areas	Moderate	[117, 138]
Mooring systems	Use eco‐friendly and flexible materials	Reduces physical damage risks, allows natural water movement, and minimizes ecosystem impacts	Ensures minimal ecological disruption	Low to moderate	[117, 139]
System monitoring	Continuous ecological monitoring	Timely detection of ecological disruptions (oxygen, temperature, nutrients)	Allows proactive management	Moderate	[139, 143]
Adaptive management	Implement adaptive management practices	Adjustments based on ongoing monitoring results to optimize ecological impacts	Enhances responsiveness to ecological shifts	Moderate	[139]
Integrated design	Eco‐integrative FPV system designs	Incorporates biodiversity‐enhancing structures, habitat‐compatible configurations	Balances energy and ecological integrity	High	[138]

These mitigation measures are important in ensuring the sustained FPV systems deployment. The monitoring and best adaptive management undertaken enable a concurrent measuring and altering of the FPV systems to create an optimal working of the systems that uphold ecological integrity. It is possible to balance renewable energy production and the maintenance of aquatic ecosystems and biodiversity through the incorporation of ecological issues into FPV planning and management.

Taken together, the environmental effects described in Sections 6.1–6.5 form an interconnected FPV–aquatic ecosystem interaction pathway. FPV deployment characteristics, particularly surface coverage ratio, array spacing, and platform configuration, initiate physical drivers such as surface shading, altered air–water heat exchange, and reduced wind‐driven mixing. These processes influence water temperature, thermal stratification, dissolved oxygen dynamics, and nutrient cycling, which in turn shape biological responses including submerged vegetation growth, phytoplankton composition, fish habitat suitability, and broader biodiversity patterns. Importantly, feedback mechanisms may amplify these effects, for example, when reduced light availability and enhanced stratification promote hypoxia, stimulate nutrient release from sediments, and increase eutrophication risk. The mitigation strategies discussed in this section represent key intervention points to moderate these feedbacks and support environmentally responsible FPV deployment.

In addition to conventional mitigation strategies, recent research highlights the growing relevance of eco‐integrative floating infrastructure concepts that may fundamentally reshape the environmental footprint of FPV systems. Such approaches emphasize flexible, hydrophilic, and low‐impact floating materials that maintain stable water–surface interactions while minimizing mechanical disturbance to aquatic environments. For example, recent advances in photothermal hydrogel‐based floating membranes demonstrate how soft, adaptable floating structures can achieve high solar utilization efficiency while preserving continuous water contact and structural flexibility. Although these systems are developed for interfacial solar evaporation rather than photovoltaic power generation, they provide valuable material‐level insights for future FPV platform designs that aim to integrate renewable energy deployment with environmentally compatible, low‐impact floating architectures [149]. Incorporating such eco‐integrative design principles could enable FPV systems to move beyond impact mitigation toward more adaptive and ecosystem‐conscious deployment strategies.

## 7 Life Cycle and Sustainability Assessment

In addition to evaluating long‐term advantages like emissions reduction, resource conservation, and general sustainability, a Life Cycle and Sustainability Assessment of FPV systems offers a comprehensive understanding of their environmental performance across all stages, from material sourcing to decommissioning [150]. Evaluating the life cycle consequences of FPV technology is crucial for advancing sustainable development goals as it becomes more popular due to its dual advantages of producing clean energy and using space efficiently [151].

### 7.1 Life Cycle Stages of FPV Systems

**FIGURE 9:**
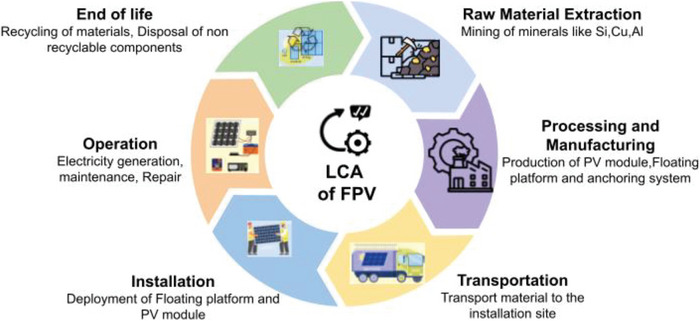
A life cycle assessment of FPV systems that highlights important phases from the extraction of materials to their disposal. Copyright The Authors, 2025.

Life cycle analysis (LCA) refers to a systematic process that assigns the environmental impacts of each of the stages of the life cycle of a particular product, and it is an effective tool in understanding how resources are utilized, and in light of emissions and the generation of waste. LCA assists in the comprehensive evaluation of FPV systems, which combine solar energy technology with floating platforms on water bodies. This evaluation covers everything from material extraction to manufacturing, transportation, installation, operation, and end‐of‐life management. Opportunities for sustainable design and management, as well as hotspots of environmental consequences, are identified by this multi‐stage investigation [152]. Since FPV technology is progressing, it may have two positive effects of both producing clean energy and using space productively. It is necessary to evaluate the life cycle effects and attribute them to sustainable development objectives [153]. The main life cycle stages and the associated environmental interactions are depicted illustratively in Figure 9.

#### 7.1.1 Raw Material Extraction

The initial phase aims at acquiring raw materials required in FPV components. Silicon is refined to high‐purity wafers used in solar cells in an energy‐intensive process derived from quartz. Aluminum, which due to its light weight use and corrosion resistance as frame and support materials, is produced through mining of bauxite and electrolysis, both of which are power‐intensive processes with large quantities of electricity and CO_2_ emissions. These include copper mining required in electrical wiring that causes considerable amounts of air and water pollution, or polymer float material HDPE that uses feedstocks to make the material based on fossil products [154]. An application such as steel in anchors and mooring also has a demand in energy. Such mining operations are significant contributors to negative environmental factors such as greenhouse emission, habitat intrusion, and depletion of resources [155].

#### 7.1.2 Material Processing and Manufacturing

In this phase, raw materials are converted into photovoltaic modules, floating platforms, electrical systems, and structural supports. Photovoltaic cell production is particularly energy‐intensive, with processes such as wafer slicing, doping, and coating generating substantial embedded carbon emissions. Fabrication of floating structures typically involves molding polymers like HDPE, which can release volatile organic compounds and generate waste. The manufacturing of inverters and electrical components also requires rare materials and produces electronic waste [156]. Water usage and chemical treatments in PV fabrication present additional environmental concerns. Some of the effects can be reduced by transitioning into renewable resources of energy in manufacturing [157].

#### 7.1.3 Transportation and Logistics

Transportation involves moving raw and finished materials across global supply chains from mining sites to factories, and finally to the FPV installation site. These logistics processes, dependent on fossil fuel‐powered vehicles (e.g., trucks, rail, ships), contribute significantly to carbon emissions, air pollution, and fuel consumption [158]. The transportation phase contributes to GHG emissions mainly due to fuel combustion in vehicles and ships. During transportation, large, heavy parts like aluminium frames and steel anchors use more fuel and produce more pollutants. Despite being effective across long distances, marine shipping includes the risk of unintentional fuel leaks and ballast water discharge, both of which may disrupt marine ecosystems. If not adequately recycled or reduced, packaging materials used to protect components during transit also contribute to trash generation.

#### 7.1.4 Installation and Commissioning

FPV systems installation assumes the implementation of floating platforms into water bodies, the usage of solar modules, the fastening of structures, and the electric assembly. Cranes, barges, and boats (heavy equipment) are used with diesel engines often and greenhouse gases and particulate matter are emitted. Despite the short duration nature of the construction, installation can create temporary imbalances in the aquatic ecosystem due to noise, water turbidity, and disturbance of sediment. The anchoring systems may affect local flora and fauna and have an effect on sediment transport, which might lead to coastal issues [159].

#### 7.1.5 Operation and Maintenance (O&M)

The operating phase, which typically lasts 20 to 30 years, is the longest and most stable stage. The electricity produced by an FPV system does not have any direct emissions, and the cooling property of water can be used to enhance panel efficiency due to financial gains on heat loss‐related inefficiencies. Depending on climatic conditions, this cooling can raise energy yield by 5%–15% relative to comparable ground‐mounted systems. Maintenance operations involve infrequent cleaning to clear dust, bird droppings, and algae and biofouling off floats, which reduce performance [160]. Fresh or recyclable water is frequently employed, and some attention to the condition should be paid to prevent the exhaustion of water resources. O&M also includes inverter repairs or changes, electrical system maintenance, and mooring changes to accommodate shifts of water level or effects of climate [161].

#### 7.1.6 End‐of‐Life (EoL) Management

Decommissioning FPV systems involves safe removal of modules, floats, anchors, and electrical components at the end of their operational lifespan. Recycling PV modules enables recovery of valuable materials such as glass, silicon wafers, aluminum frames, and rare metals, which can reduce the demand for virgin material extraction. However, the current global recycling infrastructure is limited, and many regions lack effective policies or technologies to manage solar panel waste adequately. Recycling operations might be complicated by the degradation of floating platforms composed of HDPE or other polymers during extended exposure to UV light and water. These items could be thrown away or burned if proper recycling isn't done, which would pollute the environment. Additionally, pieces of damaged floats present a risk of aquatic ecosystems becoming contaminated with microplastics, which could have long‐term ecological effects [162].

### 7.2 Comparative Environmental Footprint

FPV systems show a reduced environmental impact in a number of areas when compared to ground‐mounted PV systems. The main benefit of FPV is that they don't require clearing land, protecting terrestrial ecosystems, and removing emissions from land acquisition. Their location over bodies of water creates a cooling effect that enhances module efficiency and prolongs operational life, which lowers lifecycle emissions per kWh generated. Research suggests that when FPV systems are compared to comparable land‐based PV systems, greenhouse gas emissions may be reduced by 5%–10% [163, 164]. Environmental models and lifecycle assessment tools can be used to quantify the ecological impacts of FPV systems. Platforms such as OpenLCA, SimaPro, and RETScreen make it simple to evaluate the FPV components' recyclability, carbon footprint, and material flow. Simulation tests comparing the FPV system with one placed on the ground showed significant differences. The water‐mounted arrays have the advantage of producing an additional 20% of energy capacity and an overall improvement of over 25%. These advantages are mostly linked to improved irradiance reflection and off‐water cooling.

Furthermore, FPV may reduce water evaporation by up to 70%, which is an important co‐benefit in areas that are prone to drought and aridity [165]. However, heat stratification, possible leaching from polymer floats, and disturbance of the aquatic environment are possible hazards specific to FPV. To properly quantify ecological trade‐offs, site‐specific studies are required for these consequences. Improved energy yields and co‐benefits to the hydrosphere frequently make up for FPV systems' higher material and transport emissions, even though their installation and anchoring procedures are more complicated. From the perspective of recyclability, FPV and conventional PVs share a number of material properties, such as silicon, glass, and aluminum, all of which have a significant potential for recovery [165, 166]. However, because of contamination or degradation, special polymer‐based float structures might be difficult to recycle, highlighting the need for sustainable material innovation [167].

### 7.3 Sustainability Metrics in FPV Projects

Sustainability metrics offer a quantifiable basis for evaluating the long‐term environmental performance of FPV systems. Energy Payback Time (EPBT), the time required for a system to generate the amount of energy used in its manufacturing and deployment, is often slightly lower in FPV systems due to higher energy yields from the water‐based cooling effect [168]. Some sustainability factors, such as the following, are computed to determine the long‐term environmental and operational credentials of FPV systems in a quantitative way. The following Table 6 lists various measurements; the range of numbers is based on some recent research, and links to official sources are included for verification.

**TABLE 6:** Key sustainability metrics in FPV projects.

Metric	Description	Estimated value/range	Refs.
Energy payback time (EPBT)	Time required for the FPV system to generate equivalent energy used in production	1.0–2.8 years	[169]
Energy return on investment (EROI)	Ratio of energy produced versus energy consumed during the life cycle	≈29.8	[170]
Carbon savings	CO_2_ emissions avoided per kW of installed FPV capacity over its lifecycle	1500–2200 kg CO_2_/kW	[171]
Water evaporation reduction	Percentage decrease in reservoir water loss due to FPV coverage	60%–70% (e.g., ∼60.2%; ∼81 500 m^3^/30 years)	[172, 173]
Module efficiency gain	Increased module output attributed to water cooling	5%–15%	[174]
Lifecycle emission reduction	Total CO_2_ emissions reduction compared to land‐based PV systems	5%–10% lower	[175]
Material recyclability	Recoverable proportion of key materials (aluminum, glass, silicon) at end‐of‐life	80%–90%	[176]

These metrics highlight how FPV systems have a great sustainability profile. EPBT (Energy Payback Time) is remarkably low (in cases, lower than 3 years), with some of the lowest cases being 1 year only, while energy returned to investments (EROI) is close to 30, which is extremely high. FPV also have their value to the environment in terms of carbon economy and water savings [177]. In the long term, they are also more sustainable because of the significant number of recyclable materials and the efficiency advantages from using natural cooling. Monitoring these data will assist stakeholders in making informed decisions that will guide future policymaking and FPV deployment.

## 8 Global Deployment of FPV Systems

Regional deployment patterns of FPV systems differ significantly. In South Korea, FPV deployment has progressed beyond pilot scale, supported by strong policy incentives and the availability of large reservoir‐based sites. In contrast, European FPV development has focused primarily on pilot‐scale and demonstration projects, including hybrid FPV–hydropower applications and policy‐supported research initiatives aimed at evaluating technical feasibility, environmental compatibility, and regulatory integration.

China is spearheading large‐scale installations of FPV, which is still being developed throughout Asia. The Wenzhou Taihan Solar PV Park, which spans roughly 4.9 km^2^ and has an installed capacity of 550 MW, is located in Zhejiang Province [178, 179]. With a 320 MW capacity and an about $260 million investment, the Hangzhou Fengling Electricity Science Technology Solar Farm is situated across the Changhe and Zhouxiang reservoirs [180]. Other well‐known Chinese FPV projects include the Three Gorges Huainan Floating Solar PV Park, which produces 150 MW of power at an estimated cost of $151 million [181] and the Sungrow Huainan Solar Farm in Anhui Province, which produces 40 MW at an estimated cost of $45 million [182]. India closely follows China's progress, hosting several notable FPV initiatives. The Omkareshwar Floating Solar Park, which is being built on Madhya Pradesh's Narmada River, is around 20 km^2^ in size, has a 600 MW capacity, and costs about $409 million [183]. The NTPC Simhadri plant in Andhra Pradesh (25 MW, $17.45 million), the NTPC Kayamkulam installation in Kerala (92 MW, $58 million), and the NTPC Ramagundam Floating Solar Project in Telangana (100 MW, $56 million) are other significant Indian projects [184, 185]. Japan contributes with FPV systems that are small and effective. Models of Japan's technological accuracy and efficient use of space are the Umenoki Floating Solar Plant in Saitama Prefecture, which produces 7.5 MW, and the Yamakura Dam FPV Plant in Chiba Prefecture, which produces 13 MW [186, 187].

In Southeast Asia, Malaysia's Solarvest Selangor Floating Solar PV Park, which cost roughly $11.3 million to build, produces 13 MW of electricity. Additionally, the nation runs smaller FPV systems, including Ulu Sepri (0.27 MW) and Sungai Labu (0.108 MW) installations [188]. Some of the biggest FPV ambitions in the world are located in Indonesia. Currently in development, the Duriangkang Floating Solar System will have a capacity of 2200 MW, span around 16 km2, and cost an estimated $2.235 billion. At an approximate cost of $95 million, the Cirata Reservoir FPV Power Project is currently in operation and generates 145 MW [189, 190, 191]. With its Da Mi Floating Solar Park, which costs $66.44 million and produces 47.5 MW of sustainable energy, Vietnam also makes a contribution [192].

In South Korea, FPV projects of all sizes are integrated. These include the 280 MW Dangjin and Goheung County Floating Solar Plant, the 41 MW Hapcheon Dam Floating Solar System, which cost $65 million, and the 18.7 MW Gunsan Retarding Basin FPV Project, which cost about $24.8 million [193, 194, 195, 196]. With the Sirindhorn Dam FPV Project (45 MW, $34 million) and the Wisewood Floating PV System (1.26 MW), Thailand expands its regional portfolio [197, 198]. Among the biggest FPV systems in Southeast Asia is Singapore's Tengeh Reservoir Floating Solar Plant, which has a 60 MW capacity [198]. Thailand's 45 MW Sirindhorn Dam FPV Project ($34 million) and 1.26 MW Wisewood Floating PV System contribute to regional diversity [197, 198]. Singapore's 60 MW Tengeh Reservoir Floating Solar Plant stands as one of the largest in Southeast Asia [178].

In Europe, FPV deployment often utilizes underutilized water surfaces such as sandpits and reservoirs. The Netherlands operates the 27.4 MW BayWa‐re Floating Solar PV Park IV ($29.455 million) and the 14.5 MW Sekdoorn Floating Solar Park ($16.259 million) [195, 199]. France's 17 MW O'Mega 1 ($14.2 million) [200] and Portugal's Alto Rabagão Hybrid Dam‐FPV System demonstrate integration with existing hydro facilities [201]. The UK's notable system is the 6.36 MW Queen Elizabeth II Reservoir Solar Plant (£6.5 million) [202].

In the Americas, FPV technology is becoming more and more popular with projects like the Far Niente installation in California (0.175 MW) and the Sayreville Water Reservoir FPV System in New Jersey (4.4 MW) [203, 204]. In an effort to integrate solar energy with its current dam infrastructure, Brazil is also exploring hybrid FPV‐hydropower projects.

**FIGURE 10:**
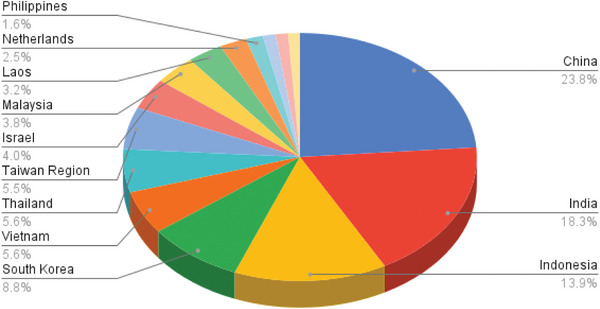
Global distribution of cumulative FPV installations by country. Copyright The Authors, 2025.

Africa's energy needs and water management goals are met by the Seychelles Floating Solar Plant on Mahé Island, which generates 4 MW of renewable energy [205]. The cumulative capacity and expected worldwide distribution of FPV installations by nation through 2031 are shown in Figure 10, which also highlights differences in scale, investment, and regional focus [206].

From the chat insight, we see that China is projected to have an installed capacity of 13 783 MWdc (23.8%) to take the lead in the number of FPV deployments by 2031. India has 10 614 MWdc (18.3%), followed by Indonesia with 8082 MWdc (13.9%). Strong growth can also be seen by South Korea (5083 MWdc, 8.8%), Vietnam (3265 MWdc, 5.6), and Thailand (3265 MWdc, 5.6). Other, smaller though significant, shares are accredited to Laos (1874 3.2% 00 md), the Netherlands (1440 2.5% 00 md), and the Philippines (917 1.6% 00 md). France (672 MWdc, 1.2%), Portugal (662 MWdc, 1.1%) and Singapore (624 MWdc, 1.1%), further diversify the global presence. The spread notes that there is increased adoption of FPV across the world, with top countries leading in terms of significant adoption and others slowly expanding their capacities.

Among the three main structural types of FPV systems, such as pontoon‐based, flexible membrane‐based, and submerged, the pontoon‐based design is the most widely adopted and technically mature. These systems use high‐density polyethylene (HDPE) pontoons to support solar panels over calm water bodies such as reservoirs and lakes. Large‐scale projects like Indonesia's Duriangkang (2200 MW), South Korea's Saemangeum (2100 MW), China's Wenzhou Taihan (550 MW), and India's Ramagundam (100 MW) highlight their global preference. With efficiency gains of 10%–15% over ground‐mounted PV and capital costs of $0.85–1.2 million per MW, pontoon‐based FPV are proving both effective and economical [42, 44, 183, 190, 198]. In addition, flexible membrane‐based systems are a newer innovation suited to offshore and wave‐exposed sites. They support lightweight, frameless PV modules with hydro‐elastic mats that flex with water movement. The 2 MW Soneva Secret FPV in the Maldives and the Ocean Sun BOOST project in Spain (275 kWp) are two examples. At $1.5–2 million per MW, they are nevertheless more costly and less scalable, despite being ideally suited for marine conditions [206]. Furthermore, submerged FPV are still in the experimental stage, their modules are made to be partially or completely submerged for storm protection and cooling. Efficiency increases of up to 20.8% have been demonstrated by the Hide & Shine pilot project in the Netherlands [207], however their scope is constrained by issues with sealing, corrosion, and maintenance. Up until now, no project has used more than a few hundred kilowatts.

Overall, pontoon‐based FPV remains the most viable for large‐scale deployment, as seen in Indonesia's Cirata Reservoir (145 MW for $95 million) and India's Ramagundam plant (100 MW for $56 million). Flexible membrane systems may find a role in offshore solar where wave resilience is key, while submerged FPV, though promising, are still at the research stage and not commercially feasible.

## 9 Limitations, Policy Landscape, and Research Gaps

Although FPV systems have significant advantages and good potential, there have been a number of constraints that remain a barrier to the implementation and sustainability of the systems. These limitations are both technical, environmental and institutional. Moreover, with no regularized policy patterns and standardization, regulatory clarity is not easily met. In order to realize the true potential of FPV, we must have an idea of the systemic bottlenecks as well as opportunities of where future research and innovation is possible.

### 9.1 Technical and Environmental Limitations

The effects of ensuring mooring and anchoring are among the technical most serious weaknesses of the FPV system, especially within deep waters and tides. Depending on the water depth and environmental loads, anchoring costs may be up to 20–25% of the overall installation costs when offshore projects are involved. Unlike land‐based solar [208], FPV arrays need to possess robust structural frameworks since the arrays are subject to dynamic water movements. Another operational challenge has been associated with improperly designed mooring systems that have been involved with platform drift, cable fatigue, and mechanical breakdown in high‐wind events [209]. Maintenance is the other operational challenge. Floating platforms can only be accessed by specialized vessels and equipment, and hence, repairs are labor‐intensive and costly. Maintenance costs are estimated to be 15%–30% higher than those of comparable PV systems on land, especially when biofouling or high humidity are present [210]. There are still issues with scaling. Despite the success of pilot and mid‐range FPV systems (1–100 MW), large‐scale deployment (100 MW and beyond) has compounding hazards related to structural flexibility, logistical, and environmental approval concerns of the power transmission. For instance, despite significant financial interest, Indonesia's 2200 MW Duriangkang FPV project is still in development due to technical and legal challenges [211]. The issue of biodiversity is becoming increasingly important from an ecological perspective. Additionally, FPV systems can reduce light penetration by up to 95%, which can disrupt trophic interactions and prevent photosynthesis in aquatic environments. Long‐term shade has been shown to lower the macrophyte density in the covered areas by 60%–80% [212]. Additionally, a damaged polymer float may produce microplastics, which could alter the water chemistry and benthic ecosystem, and anchoring may disturb the sediment [213].

### 9.2 Policy and Standardization Issues

There are currently no worldwide standards and a fragmented policy environment for FPV systems. It is more difficult to legalize FPV since many countries still view it as a combination of offshore infrastructure and land‐based PV. The second issue is that FPV developers in India must navigate two levels of regulation by the energy and water resource ministries, which increases the average project implementation time by 612 months [214]. The performance and safety of FPV are not covered by any rules. Most FPV systems are installed using modified standards that are intended to install PV systems on offshore wind platforms or the ground, which may not be appropriate for the floating interface [215]. Important factors like cable waterproofing, buoyancy testing, structural certification, and safety distances for recreational water bodies are all impacted by this lack of harmonization. Ecological risks are also assessed inconsistently due to the lack of standardization in environmental impact assessments (EIAs) for FPV. While other nations, such as Japan and the Netherlands, have created stronger FPV‐specific EIA procedures, they are still the exception rather than the rule [216].

### 9.3 Identified Gaps and Future Research Directions

There are still significant gaps in our understanding of FPV's long‐term environmental consequences. The majority of ecological assessments are conducted over a brief time frame (one to three years) and do not address concerns about how these ecological parameters affect nutrient cycling, sedimentation, and species adaption. Longitudinal studies (10–15 years) are required to investigate this issue since it is unable to incorporate the investigation of multi‐use platforms, such as FPV in aquaculture, desalination, or hydrogen generation [217]. Pilot studies suggest that hybrid FPV‐hydrogen systems could significantly improve energy utilization by storing excess solar electricity as hydrogen gas. For instance, according to a hypothetical design proposed by [218], FPV‐coupled electrolysis could reduce hydrogen production costs by 18%–22% when compared to PV systems on land because of improved cooling and space efficiency. Furthermore, digital twins of FPV and data‐driven modeling have not yet been created. Only a few simulation systems, such as PVsyst and SAM, have been specifically built to be calibrated with floating installations [219]. Predictive maintenance and optimized performance at scale should be achieved by investigating AI‐driven monitoring systems that incorporate hydrological, electrical, and ecological data. To synthesize and benchmark existing FPV review and case‐study literature across technological design, performance, ecological interactions, and sustainability dimensions, a consolidated comparative overview is provided in Table 7.

**TABLE 7:** Comparison of FPV review and case‐study literature across technology design, performance, environmental interactions, and sustainability dimensions (✔ = explicitly addressed; △ = partially addressed; **×** = not addressed).

Refs.	FPV technology classification	Engineering design and mooring analysis	Quantitative performance comparison	Aquatic ecosystem impact mechanisms (DO, temp, PAR)	Integrated FPV–ecosystem conceptual framework	Life cycle and sustainability metrics (EPBT, CO_2_, water)	Policy, regulation and deployment barriers	Case studies and global deployment analysis
[52]	✔	✔	✔	△	**×**	**×**	✔	✔
[91]	✔	✔	△	△	**×**	**×**	△	✔
[109]	✔	✔	✔	△	**×**	△	✔	✔
[163]	✔	✔	✔	△	**×**	△	△	✔
[220]	✔	✔	✔	△	**×**	△	△	✔
[221]	✔	✔	✔	✔	**×**	✔	△	✔
[222]	✔	△	✔	**×**	**×**	**×**	**×**	△
[223]	✔	✔	✔	✔	**×**	✔	✔	✔
[224]	✔	✔	✔	△	△	**×**	✔	△
[225]	✔	✔	✔	✔	**×**	△	△	✔
[226]	✔	✔	✔	△	**×**	△	△	✔
[227]	✔	✔	✔	✔	**×**	**×**	△	✔
[228]	✔	✔	△	△	**×**	△	✔	△
[229]	✔	✔	△	**×**	**×**	**×**	**×**	△
This paper	✔	✔	✔	✔	✔	✔	✔	✔

This table presents a structured comparison of existing FPV‐related review and case‐study literature based on a common set of technical, environmental, and sustainability criteria. The comparison shows that most previous studies focus primarily on FPV technology classification, engineering design aspects, and performance improvement. However, fewer studies work on aquatic ecosystem impact mechanisms, life‐cycle sustainability indicators, and policy or regulatory issues within a single analytical framework. In contrast, this study integrates FPV system design, quantitative performance characteristics, ecosystem interactions, sustainability considerations, and deployment challenges into a unified FPV–ecosystem perspective. This integrated approach addresses important gaps in the existing literature and provides a comprehensive basis for future research and practical implementation of FPV systems.

## 10 Conclusions

Floating photovoltaic systems have swiftly emerged as a cutting‐edge substitute and an essential part of the worldwide renewable energy grid. As this analysis shows, FPV exploits underutilized water surfaces to generate clean power, suggesting that there are other ways to address land scarcity, energy consumption, and climate mitigation. By classifying FPV technologies into pontoon‐based, flexible membrane, and submerged technologies—each with unique benefits and drawbacks—this research illustrates the adaptability of FPV to a variety of geographic, environmental, and economic contexts. The most traditional and widely used systems are pontoon‐based, whereas flexible membrane and submerged systems continue to have difficulty with scalability and durability. Water‐based cooling, tilt angle, spacing, elevation, and material selection are all optimized to help boost the energy yield and ensure engineering performance stability. Digital twins, AI‐based monitoring, and auxiliary cooling and cleaning systems increase the system's long‐term dependability and efficiency.

From an engineering perspective, the proximity of FPV systems to water bodies can provide passive cooling that reduces module operating temperatures and contributes to moderate energy yield improvements compared to ground‐mounted photovoltaic systems. Reported performance gains are highly dependent on FPV configuration, system design, and climatic conditions, with more pronounced benefits observed in hot and humid environments, while temperate regions typically exhibit smaller gains. Additionally, performance depends on proper height, tilt, and spacing, all of which are supported by efficient mooring and material selection. Digital twins and intelligent monitoring support long‐term dependability and operational efficiency with additional cooling and cleaning systems. FPV installations offer an environmental impact that includes quick energy payback periods, up to 30% fewer lifecycle CO_2_ emissions, 60%–70% water savings, and excellent recyclability of most materials. FPV installations also have ecological implications, including as a 95% decrease in photosynthetically active radiation, a 1.117 mg/L drop in dissolved oxygen, and the potential for eutrophication.

Despite its great promise, FPV adoption is hampered by issues with polymer recyclability, long‐term ecological uncertainty, inconsistent laws, and a lack of uniform rules. Multifunctional benefits can be unlocked by filling in these gaps and integrating hydropower, aquaculture, and green hydrogen in a hybrid manner. FPV offers a scalable, sustainable route to climate mitigation, water‐energy co‐benefits, and fair access to clean energy with appropriate engineering optimization and ecological safeguards.

## Funding

This research received no external funding.

## Conflicts of Interest

The authors declare no conflicts of interest.

## Data Availability

This study is a review article and does not generate new experimental or simulation data. All data, figures, and tables are derived from previously published and publicly available sources, which are cited in the manuscript. Reproduced or re‐plotted materials follow applicable open‐access licenses, with corresponding copyright and license information provided in the figure and table captions.
